# Machine learning for biomarker-driven thalassemia diagnosis: a framework for causal effect estimation

**DOI:** 10.3389/fmed.2026.1926776

**Published:** 2026-10-29

**Authors:** Nora ElRashidy, M. A. Elsabagh, Esraa Hassan, Ahmed F. El Fouhil, Mohamed Y. Alhussainy, Saad Ali S. Aljohani, Ikhlas A. Sindi, Shaimaa M. Hassan, Nevein M. Abdelhady

**Affiliations:** 1 Department of Computer Science, University College in Umluj, University of Tabuk, Umluj, Saudi Arabia; 2 Faculty of Artificial Intelligence, Department of Machine Learning and Information Retrieval, Kafrelsheikh University, Kafrelsheikh, Egypt; 3 Department of Anatomy, College of Medicine, King Saud University, Riyadh, Saudi Arabia; 4 Department of Pharmacology, Al-Ghad International College for Applied Medical Sciences, Al-Madinah Al-Munawara, Saudi Arabia; 5 Department of Basic Medical Sciences, Al-Rayan National College of Medicine, Al-Rayan National Colleges, Al Madinah Al Munawwarah, Saudi Arabia; 6 Department of Biotechnology, Faculty of Science, King Abdulaziz University, Jeddah, Saudi Arabia; 7 Basic Medical Sciences Department, College of Pharmacy & Applied Medical Sciences, Dar Al Uloom University, Riyadh, Saudi Arabia; 8 Department of Clinical Pharmacy, Al-Rayan National College of Health Sciences, Al-Rayan National Colleges, Al Madinah Al Munawwarah, Saudi Arabi

**Keywords:** AI clinical decision support, biomarkers, causal discovery, counterfactual inference, hematological parameters, hemoglobinopathies, ML, molecular diagnosis

## Abstract

Thalassemia is one of the most heterogeneous diseases, with more than a thousand mutation types recorded worldwide. Molecular diagnosis of thalassemia by conventional PCR-based DNA analysis is time- and resource-consuming owing to the phenotype variability, disease complexity, and molecular diagnostic test limitations. Thalassemia diagnosis relies heavily on complex interactions among hematological biomarkers, making it challenging to distinguish causal relationships from mere statistical correlations. To address this limitation, this study proposes a causal machine learning framework that integrates causal discovery and counterfactual inference to identify biomarker-driven treatment effects for thalassemia diagnosis. The proposed approach models clinical variables using a Directed Acyclic Graph (DAG) to capture underlying causal dependencies and enables robust estimation of treatment effects while accounting for confounding factors. To address the primary objective from complementary causal perspectives, the framework combines adjusted regression for confounding control, inverse probability weighting for propensity-based estimation, Random Forest-based analysis for nonlinear feature importance and heterogeneous treatment effects, and bootstrap resampling for uncertainty quantification. The adjusted linear regression model yielded a conservative causal effect estimate of 0.3008, while propensity-based methods demonstrated stronger effects, with IPW producing an estimate of 1.0604. The bootstrap approach confirmed the stability of these findings with a 95% confidence interval of [0.8806, 1.2161], indicating statistically significant treatment influence. Furthermore, heterogeneous effect analysis using CATE revealed substantial variability across individuals, with estimates ranging between 0.0848 and 2.5200, highlighting the importance of personalized assessment. Stratified analysis across age groups showed distinct differences, with the highest effect observed in adolescents (1.3484) and lower effects in children (0.8856), emphasizing demographic-specific variations. The results demonstrate that key biomarkers, particularly hemoglobin-related features, exert significant causal influence on thalassemia diagnosis.

## 1 Introduction

Recent advances in artificial intelligence (AI) and machine learning (ML) have significantly transformed the healthcare domain by enabling accurate disease prediction, risk stratification, and clinical decision support (1–3). Predictive analytics based on clinical records, complete blood count (CBC) parameters, biochemical biomarkers, and electronic health records have demonstrated strong potential in detecting cardiovascular diseases, hematological disorders, and toxic exposure conditions at early stages. Traditional diagnostic approaches often depend on manual interpretation and expert knowledge, which may be time-consuming, subjective, and prone to variability across healthcare environments. Consequently, intelligent predictive systems have emerged as promising solutions for improving diagnostic efficiency, reliability, and accessibility (4, 5). Thalassemia is a hereditary blood disorder characterized by abnormal hemoglobin production, leading to anemia and a range of clinical complications that vary in severity from mild to life-threatening conditions (6–8). Early and accurate diagnosis is essential for effective treatment planning and long-term disease management. Thalassemia and hemoglobinopathies are genetic disorders caused by gene defects that hinder the normal production of hemoglobin, the main protein found in RBCs responsible for binding and carrying oxygen-RBCs from the lungs to the various body tissues. Normal adult hemoglobin consists of two pairs of α and β-chains, respectively. Synthesis of these proteins is coordinated to ensure equal production levels in erythropoietic cells (6). When one or both copies fail to produce normal β-globin, the α-globin gene continues its normal α-globin production. The consequence of these mutations is an imbalance of α/β-globin chain synthesis, evidently in homozygous forms, leading to the accumulation of free α-globin chains forming highly toxic aggregates (7). Both alpha (α)- and beta (β)-thalassemia phenotypes are classified separately. α-thalassemia is classified into four phenotypes: the silent carrier, trait, HbH, and Hb Bart's. Meanwhile, β-thalassemia is classified into four phenotypes: silent carrier (β++), carrier, intermedia, and major. β-thalassemia minor or trait refers to carriers of a β-thalassemia mutation with a normal β-globin on the other allele. They are clinically asymptomatic. β-thalassemia major is characterized by infancy-onset severe anemia that requires lifelong blood transfusion for survival (8). In β-thalassemia major, the excess unpaired α-globin chains aggregate to form inclusion bodies that damage RBC membranes, causing intravascular hemolysis, damage and apoptosis of erythroid precursors, and ineffective erythropoiesis (9). β-thalassemia intermedia is defined as having an intermediate thalassemia condition between minor and major. Most patients with β-thalassemia intermedia are homozygotes or compound heterozygotes for β-thalassemia. Rarely, a simple carrier of β-thalassemia can be symptomatic, such as in the case of co-inheritance of segmental duplication of the α-chain, which increases the imbalance ratio of β- and α-tetramer, resulting in a more severe phenotype (10, 11). Accurate diagnosis of thalassemia remains a critical clinical challenge due to the genetic heterogeneity and phenotypic variability of the disease (12). Conventional diagnosis typically begins with hematological screening using complete blood count (CBC) indices, including hemoglobin concentration, mean corpuscular volume (MCV), mean corpuscular hemoglobin (MCH), and red blood cell count, followed by confirmatory hemoglobin analysis using electrophoresis or high-performance liquid chromatography (HPLC) (13). However, these traditional approaches may fail to identify silent carriers, rare variants, or complex genotypes, particularly in populations with high genetic diversity (14). Recent advances in molecular diagnostics, including next-generation sequencing (NGS), long-read sequencing, and third-generation sequencing technologies, have significantly improved diagnostic accuracy by enabling comprehensive detection of point mutations, copy number variations, and complex structural abnormalities within globin gene clusters (15). Molecular Diagnosis characterization now plays an increasingly important role in identifying rare and complex thalassemia variants that may be missed by standard screening methods (16). In parallel, emerging evidence suggests that integrating hematological biomarkers with genetic and Molecular Diagnosis data can improve diagnostic precision, support early disease stratification, and facilitate personalized therapeutic decision-making (12, 15). These advances provide a strong clinical foundation for applying causal inference methods to better understand biomarker-driven diagnostic pathways and treatment-related outcomes in thalassemia. Consequently, there is a growing need for intelligent data-driven systems that not only predict disease outcomes but also uncover the underlying causal mechanisms driving thalassemia progression. Recent advances in machine learning have demonstrated significant potential in medical diagnosis, particularly in handling high-dimensional clinical data (17). However, most existing approaches are predominantly predictive rather than causal, limiting their ability to support clinical decision-making and treatment planning. In healthcare applications, understanding *why* a prediction is made is as important as the prediction itself. This limitation has motivated the integration of causal discovery and counterfactual inference into machine learning frameworks, enabling models to move beyond correlation and identify meaningful cause–effect relationships. In this study, we propose a causal machine learning framework for thalassemia diagnosis, focusing on identifying biomarker-driven treatment effects through causal discovery and counterfactual reasoning. The proposed approach leverages clinical features such as hemoglobin levels, red blood cell indices, and demographic variables to construct a causal structure that explains disease progression. By modeling Directed Acyclic Graphs (DAGs) and estimating treatment effects, the system can quantify how specific biomarkers influence diagnostic outcomes and to simulate alternative scenarios through counterfactual inference (18, 19). The framework integrates multiple analytical components, including causal graph modeling, propensity score-based estimation, and heterogeneous treatment effect analysis, to provide a comprehensive understanding of thalassemia dynamics. For instance, HbA2 is treated as a key explanatory variable, enabling the estimation of its causal effect on diagnosis while accounting for confounding factors such as age, gender, and hematological parameters. This allows the system to distinguish between correlation and causation, thereby improving interpretability and clinical relevance. Furthermore, the proposed approach incorporates advanced machine learning techniques, including Random Forest-based feature importance analysis and Conditional Average Treatment Effect (CATE) estimation, to capture nonlinear relationships and patient-specific variations. These capabilities enable the identification of subgroups that may respond differently to treatment, supporting personalized medicine strategies. The contributions to this work can be summarized as follows:

1. A novel integration of causal discovery and counterfactual inference for thalassemia diagnosis.
2. A biomarker-driven analysis framework that identifies key factors influencing disease outcomes.
3. A hybrid methodology combining statistical causal inference with machine learning models for improved interpretability and predictive performance.

### 1.1 Related works

Recent advances in artificial intelligence (AI) and machine learning (ML) have significantly transformed the healthcare domain by enabling accurate disease prediction, risk stratification, and clinical decision support. Predictive analytics based on clinical records, complete blood count (CBC) parameters, biochemical biomarkers, and electronic health records have demonstrated strong potential in detecting cardiovascular diseases, hematological disorders, and toxic exposure conditions at early stages. Traditional diagnostic approaches often depend on manual interpretation and expert knowledge, which may be time-consuming, subjective, and prone to variability across healthcare environments. Consequently, intelligent predictive systems have emerged as promising solutions for improving diagnostic efficiency, reliability, and accessibility that are illustrated in Table 1. Bhagya et al. (20) created predictive models for these complications using data from 357 patients, employing Random Forest, XGBoost, and a Neural Network in PyTorch. The Neural Network yielded superior performance in both cases, supported by Explainable AI methods like SHAP for identifying key risk factors and LIME for case-specific insights. Key predictors identified include hypogonadism, serum ferritin, hemoglobin, ALT, and AST. The findings underscore the potential of Explainable AI in enhancing risk assessment and clinical decision-making in thalassemia management. Alan et al. (21) conducts a systematic performance analysis of various machine learning algorithms for early heart disease detection using clinical data from the Kaggle Heart Failure dataset. It evaluates tuned Random Forest, tuned XGBoost, and a soft voting ensemble with a consistent preprocessing and validation protocol. Results indicate moderate accuracy but strong ROC–AUC performance and balanced sensitivity-specificity, underscoring the need for multi-metric evaluation in clinical settings. This research offers a rigorous framework for objective assessment and model selection in heart disease prediction. Yonathan et al. (22) suggest a cross-sectional study involving 54 males and 214 females with microcytic hypochromic anemia developed two new formulas based on Mean Corpuscular Hemoglobin Concentration (MCHC). The first formula, derived from binary logistic regression, is expressed as 21.33 + 0.9MCH − 1.3MCHC, while the second is the ratio of Mean Corpuscular Volume (MCV) to MCHC squared multiplied by 100 (MCV/MCHC² × 100). These new formulas exhibited higher specificity (92.50% and 90.01%), positive predictive value (93.48% and 91.55%), negative predictive value (85.38% and 85.71%), accuracy (89.55% and 88.81%), Youden Index (79.66% and 77.85%), and Area Under the Curve (AUC) (0.932 and 0.934) compared to established erythrocyte indices, although the Shine and Lal Index achieved the highest sensitivity (98.65%). Sekaran et al. (23) suggest a binary classification model using eXtreme Gradient Boosting (XGB) was developed to differentiate normal individuals from those with ID, hemoglobinopathies, or other anemias, trained on 5,520 complete blood count (CBC) results and validated on 2,367 results. An independent dataset of 64,100 CBC results was analyzed, predicting 40,300 individuals (62.9%) as normal. The XGB model achieved an area under the ROC of 0.97. Reference limits derived via the refineR algorithm from this subset closely matched directly obtained values, particularly enhancing accuracy for hematocrit, hemoglobin, and red cell concentrations. This approach enabled the creation of sex- and age-specific ranges, addressing limitations of direct methodologies. Shaat et al. (24) reveal variable performance of complete blood count (CBC)-derived indices in clinical settings. This commentary suggests a diagnostic framework that combines CBC indices, iron-related biomarkers, and confirmatory testing to improve diagnostic accuracy and equity in various healthcare environments. Mishra et al. (25) introduces an optimized stacking ensemble model that integrates Random Forest, XGBoost, and CatBoost as base models with a Logistic Regression meta-model, enhancing predictive accuracy. Tested on the Heart Statlog Hungary Cleveland dataset, encompassing 1,190 samples and 14 clinical features, the model underwent extensive preprocessing, including treating missing data and identifying outliers. Hyperparameters were fine-tuned using grid search, and model robustness was evaluated across various train-test splits and through cross-validation. The stacking model achieved a maximum accuracy of 97.98%, precision of 98.57%, recall of 95.83%, F1-score of 97.18%, and AUC-ROC of 98.64%, demonstrating superior predictive reliability compared to individual models. This approach offers a practical decision-support system for early CVD diagnosis. Jaiswal et al. (26) develops a robust machine learning system using Gradient Boosting algorithms (XGBoost, LightGBM, CatBoost) combined in a Stacking Ensemble, with Logistic Regression as a meta-learner. Data from the Cleveland, Hungary, and Statlog datasets were preprocessed using SMOTE, and hyperparameters were optimized via Randomized Search CV. The model achieved 93.28% accuracy, 95.08% precision, 92.06% recall, 93.55% F1-score, and a ROC-AUC of 0.9705. Key features for prediction included age, cholesterol level, blood pressure, and smoking status, supported by SHAP for interpretability. The model is deemed effective for predicting heart disease, showcasing robustness and generalizability. Lu et al. (27) addresses the challenges of assessing occupational lead exposure by developing a novel medical prior-informed ACO-RF model. This model, designed to incorporate lead's Absorption-Distribution-Metabolism-Excretion (ADME) pathways, significantly improve predictions of blood lead (BL) and urine lead (UL) levels. Validated on a multicenter cohort of 2,867 exposed workers in Jiangsu Province, China, the model reduced feature dimensionality by over 75% and achieved high predictive performance with AUC-ROC values of 0.9646 for BL and 0.9319 for UL. It also improved the recall of abnormal BL samples from 0.409 to 0.841, effectively decreasing missed diagnoses of subclinical exposure. This framework serves as an interpretable tool for early lead exposure risk stratification and contributes to machine learning applications in environmental and occupational health research. Sun et al. (28) examines advances in artificial intelligence and machine learning for heart disease prediction, highlighting three main model types: Gradient Boosted Trees, Transformer-Based Sequence Models, and Artificial Neural Networks. Each method offers distinct advantages; Gradient Boosted Trees excel with structured data and provide clear feature importance, Transformer models effectively analyze large electronic health records to identify long-term patterns, while Artificial Neural Networks adeptly capture complex relationships. The review discusses datasets, data preparation, and evaluation metrics such as accuracy and F1-score. However, challenges remain for clinical implementation, including the need for model transparency, reduced algorithmic bias, integration into medical workflows, and robust data privacy protections.

**Table 1:** The related work summarization.

Ref.	Study	Dataset/samples	Methodology	Task	Best results	Key Contributions
(20)	Bhagya et al.	357 patients	Random Forest, XGBoost, Neural Network (PyTorch), SHAP, LIME	Prediction of thalassemia-related complications	Neural Network achieved the best performance	Identified key predictors such as hypogonadism, serum ferritin, hemoglobin, ALT, and AST; integrated
(21)	Alan et al.	Kaggle Heart Failure dataset	Tuned Random Forest, Tuned	Early heart disease detection	Strong ROC–AUC with	Proposed rigorous multi-metric
(22)	Yonathan et al.	54 males and 214 females with microcytic	Binary Logistic Regression, Novel	Differentiation of anemia conditions	AUC = 0.932 and 0.934	Developed two new diagnostic formulas
(23)	Sekaran et al.	5,520 CBC training results, 2,367 validation results, 64,100 independent CBC results	eXtreme Gradient Boosting (XGB), refineR algorithm	Classification of anemia and hematological conditions	AUC-ROC = 0.97	Created sex- and age-specific reference ranges and improved diagnostic reliability
(24)	Shaat et al.	Clinical CBC-derived indices	Diagnostic framework combining CBC indices and biomarkers	Improved anemia diagnosis	Variable diagnostic performance discussed	Proposed integrated framework combining biomarkers and confirmatory tests for equitable diagnosis
(25)	Mishra et al.	Heart Statlog Hungary Cleveland dataset (1,190 samples, 14 features)	Stacking Ensemble (RF, XGBoost, CatBoost + Logistic Regression meta-model)	Cardiovascular disease prediction	Accuracy = 97.98%, AUC-ROC = 98.64%	Demonstrated superior predictive reliability using optimized stacking ensemble
(26)	Jaiswal et al.	Cleveland, Hungary, and Statlog datasets	Gradient Boosting (XGBoost, LightGBM, CatBoost), Stacking Ensemble, SHAP	Heart disease prediction	Accuracy = 93.28%, ROC-AUC = 0.9705	Combined boosting algorithms with interpretability for robust prediction
(27)	Lu et al.	2867 workers exposed to lead (China)	Medical prior-informed ACO-RF model	Blood and urine lead exposure prediction	AUC-ROC = 0.9646 (BL), 0.9319 (UL)	Improved recall of abnormal blood lead samples and reduced missed diagnoses
(28)	Sun et al.	Review of AI/ML heart	Gradient Boosted Trees	Heart disease prediction review	Comparative analysis of AI models	Discussed challenges of transparency

Several recent studies have explored different ML and deep learning techniques for disease prediction and medical diagnosis. Bhagya et al. (20) utilized Random Forest, XGBoost, and Neural Networks combined with Explainable AI methods such as SHAP and LIME to predict thalassemia-related complications, highlighting the importance of interpretable medical AI. Alan et al. (21) investigated ensemble learning approaches for heart disease prediction using the Kaggle Heart Failure dataset and demonstrated the importance of multi-metric evaluation frameworks. Similarly, Mishra et al. (25) and Jaiswal et al. (26) proposed advanced stacking ensemble architectures integrating Gradient Boosting models to improve cardiovascular disease prediction accuracy and robustness. In the field of hematological analysis, Yonathan et al. (22) introduced novel MCHC-based diagnostic formulas for differentiating anemia conditions, while Sekaran et al. (23) developed an XGBoost-based classification model using large-scale CBC datasets for identifying anemia and hemoglobinopathies. Moreover, Lu et al. (27) proposed a medical prior-informed ACO-RF framework for predicting blood and urine lead exposure levels, demonstrating the effectiveness of interpretable AI in occupational health applications. Additionally, Sun et al. (28) reviewed the recent evolution of AI techniques in heart disease prediction, emphasizing the growing role of Gradient Boosted Trees, Transformer-based models, and Artificial Neural Networks in clinical decision-making systems. Despite these promising advancements, several limitations remain regarding model interpretability, generalization capability, dataset imbalance, and clinical integration. Therefore, further research is required to develop robust, explainable, and scalable AI-driven diagnostic systems capable of achieving high predictive performance while maintaining transparency and reliability in real-world healthcare applications.

## 2 Methods and materials

### 2.1 Overview of the proposed framework

We present a causal machine learning framework for thalassemia diagnosis that combines causal discovery, statistical causal inference, and machine learning-based heterogeneous treatment effect modeling to reveal biomarker-driven diagnostic effects, as shown in Figure 1. The framework is designed to transcend the conventional correlation-based prediction models by explicitly modelling causal dependencies between hematological biomarkers and demographic variables (29, 30). The proposed methodological approach is based on the use of Directed Acyclic Graphs (DAGs), propensity-based causal estimation, bootstrap uncertainty analysis and Conditional Average Treatment Effect (CATE) estimation to quantify the influence of biomarkers on thalassemia diagnosis and severity. The DAG was specified as *a priori* using established clinical knowledge regarding hematological indices and thalassemia diagnosis. Observed correlations were used to characterize the data structure but were not treated as evidence of causal direction. The adjustment set was selected to block plausible backdoor paths between HbA2 exposure and diagnosis while avoiding adjustment for variables considered potential mediators or consequences of the diagnostic process. The workflow of the proposed framework includes six major stages: (i) Clinical data collection and preprocessing. (ii) Feature encoding and normalization. (iii) Modeling of dependencies and building of causal graph. (iv) Multiple causal inference methods for treatment effect estimation. (v) Analysis of feature importance and heterogeneous effects. (vi) Sensitivity analysis and uncertainty quantification.

**Figure 1:**
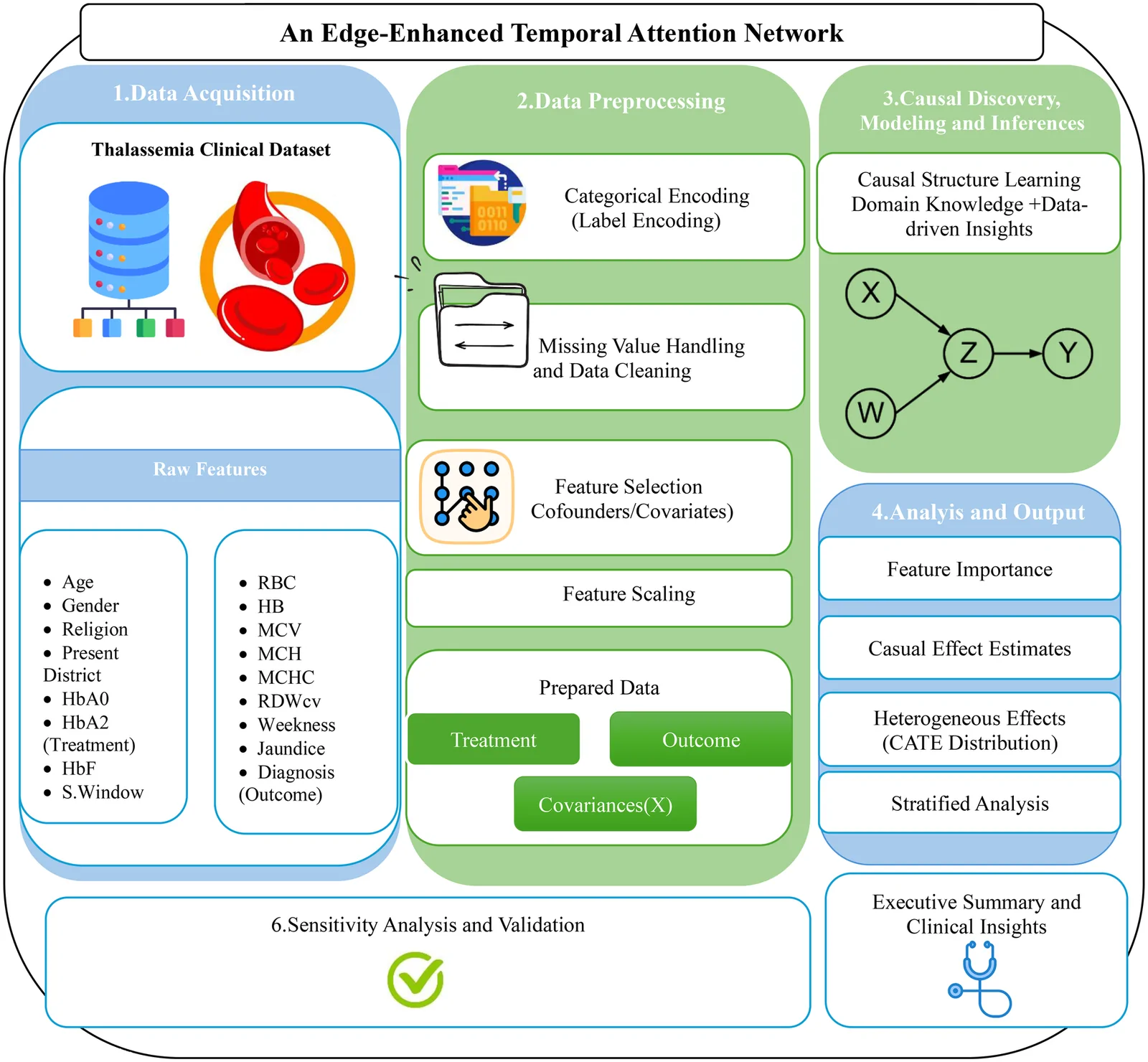
Architecture of the proposed causal machine learning framework for thalassemia diagnosis.

The proposed framework uses HbA2 as the treatment variable because of its well-established clinical significance for thalassemia diagnosis. The outcome variable is the diagnosis category. Demographic and hematological parameters are included as covariates to adjust for confounding.

### 2.2 Data preprocessing

The preprocessing phase is aimed to ensure data consistency, noise reduction and dataset preparation for causal modeling and machine learning analysis. The preprocessing pipeline includes categorical encoding, feature cleaning, normalization and covariate preparation (31–33). The categorical variables such as gender, religion, district, level of weakness, jaundice condition, and diagnosis class were converted into numerical variables using Label Encoding. This transformation allows machine learning algorithms to process categorical information efficiently while preserving the distinction between categories. The standardized representation is calculated as in Equation 1:

$$x_{\mathrm{encoded}\;}=f(x_{\mathrm{categorical}\;})$$ (1)

Where $x_{\mathrm{categorical}\;}$ is the original categorical feature, $x_{\mathrm{encoded}\;}$ is the transformed numerical representation, and f(⋅) represents the label encoding transformation. The framework identifies three main groups of variables: (i) Treatment Variable: Diagnosis Outcome Variable: HbA2. (ii) Covariates: Age, Gender, HbA0, HbF, RBC, HB, MCV, MCH, MCHC, RDWcv. (iii) Covariates were selected because they were potentially related to both the treatment and outcome variables, and therefore potentially confounders.

We used StandardScaler for feature standardization to ensure numerical stability and equal contribution of features in the training of the model. The standardized representation is calculated as in Equation 2:

$$z_{i}=\frac{x_{i}-\mu }{\sigma }$$ (2)

Where $x_{i}$ is the original feature value, μ is the feature mean, σ is the standard deviation, and $z_{i}$ is the normalized feature value. Normalization alleviates the scale differences between biomarkers and stabilizes causal estimation and Random Forest learning.

### 2.3 Proposed causal machine learning framework for thalassemia diagnosis

Exploratory data analysis (EDA) was performed to explore the relationships between biomarkers and to discover preliminary diagnostic patterns. To quantify pairwise linear associations between variables, the correlation matrix was computed. The Pearson correlation coefficient between two variables, X and Y. The standardized representation is calculated as in Equation 3:

$$r=\frac{\sum_{i=1}^{n}\;(x_{i}-\bar{x})(y_{i}-\bar{y})}{\sqrt{\sum_{i=1}^{n}\;{\left(x_{i}-\bar{x}\right)}^{2}\sum_{i=1}^{n}\;{\left(y_{i}-\bar{y}\right)}^{2}}}$$ (3)

where: *r* is the Pearson correlation coefficient, $x_{i}$ and $y_{i}$ are sample observations, and $\bar{x}$ and $\bar{y}s$ are variable means. These analyses provide an initial understanding of biomarker interactions before causal modelling.

A Directed Acyclic Graph (DAG) was constructed to represent the causal relationships between clinical biomarkers and diagnostic outcomes. The DAG encodes the variables as nodes and causal dependencies as directed edges. The graph can be formally written as in Equation 4:

G=(V,E) (4)

where: *V* denotes the set of variables (nodes), and *E* denotes the set of directed causal edges. The causal structure enables the framework to differentiate between direct effects and indirect or confounding associations, thereby improving interpretability and clinical reliability. The standardized representation is calculated as in Equation 5: The causal effect of the HbA2 on the diagnosis of thalassemia was quantified using several causal inference approaches. The crude treatment effect was estimated by a simple linear regression model. The standardized representation is calculated as in Equation 5:

$$Y=\beta_{0}+\beta_{1}T+\epsilon$$ (5)

where: *Y* represents the diagnosis outcome, *T* represents HbA2, $\beta_{1}$ denotes the estimated treatment effect, and ϵ is the residual error term. To account for confounding factors, an adjusted regression model with covariates. The standardized representation is calculated as in Equation 6:

$$Y=\beta_{0}+\beta_{1}T+\overset{n}{\underset{i=1}{\sum }}\;\beta_{i}X_{i}+\epsilon$$ (6)

where: $X_{i}$ represents the $i^{th}$ covariate, and $\beta_{i}$ represents the corresponding regression coefficient. This model estimates the causal effect of HbA2 while controlling demographic and hematological confounders.

To reduce treatment selection bias, we conducted propensity score modeling with logistic regression. The propensity score is the probability of receiving treatment conditional on observed covariates. The standardized representation is calculated as in Equation 7:

e(X)=P(T=1∣X) (7)

where: e(X) is the propensity score, *T* is the treatment assignment, and *X* represents the covariate set. Then the average treatment effect was estimated using Inverse Probability Weighting (IPW) that standardized representation is calculated as in Equation 8:

$$ATE=\frac{1}{N}\overset{N}{\underset{i=1}{\sum }}\;\left(\frac{T_{i}Y_{i}}{e(X_{i})}-\frac{(1-T_{i})Y_{i}}{1-e(X_{i})}\right)$$ (8)

This approach relieves the confounding bias and improves the robustness of causal estimation.

To estimate the Conditional Average Treatment Effect (CATE), we used Random Forest regression. The model uses the reduction in impurity across decision trees to determine feature importance. The importance of feature j is calculated as in Equation 9:

$$FI_{j}=\frac{1}{M}\overset{M}{\underset{m=1}{\sum }}\;\Delta I_{\;j,m}$$ (9)

where: $FI_{j}$ denotes the importance of feature *j*, *M* is the number of trees, and $\Delta I_{\;j,m}$ represents impurity reduction contributed by feature *j* in tree *m*. The analysis identified HB, MCV, Age, HbA0, and RBC as the most influential features.

To estimate the Conditional Average Treatment Effect (CATE) and account for heterogeneous treatment effects across individuals, we used Random Forest regression. The CATE is mathematically defined as in Equation 10:

CATE(x)=E[Y(1)−Y(0)∣X=x] (10)

where: Y(1) denotes the potential outcome under treatment, Y(0) denotes the potential outcome without treatment, and X=x represents an individual patient profile. This analysis enables individual assessment by identifying subgroups of patients with heterogeneous biomarker-driven effects.

Stratified analysis was performed to evaluate treatment effects by age groups: Child, T Adolescent, and Adult. To evaluate the differential impact of biomarkers across populations, the treatment effect was estimated separately in each subgroup. “The results showed that adolescents” had the highest causal effect, suggesting demographic heterogeneity in disease manifestation. We conducted a bootstrap-based sensitivity analysis to explore the robustness and stability of the estimates of causal effects. Bootstrap resampling draws many samples from the dataset with replacement and recomputes treatment effects. The bootstrap confidence interval is estimated by Equation 11:

$$CI_{95\text{\%}}=[Q_{0.025},Q_{0.975}]$$ (11)

where: $Q_{0.025}$ and $Q_{0.975}$ correspond to the lower and upper quantile of the bootstrap estimates. The confidence interval so obtained shows the statistical stability of the causal effects and verifies the reliability of the proposed framework. Algorithm 1 provides an intuitive procedural description of the end-to-end workflow of the proposed framework. Causal graph specification. A variable-level causal working DAG was constructed for the HPLC-based thalassemia screening dataset. The graph was specified using observed data relationships together with clinically plausible ordering constraints, because the dataset is cross-sectional and therefore cannot independently identify temporal causal direction. Age, Gender, HPLC biomarkers, hematological measurements, clinical indicators, HbA2, and Diagnosis were represented as nodes. HbA2 was designated as the primary exposure variable and Diagnosis as the outcome. Age and Gender were treated as candidate pre-exposure confounders, while HbA0 was represented upstream of HbA2 because of its strong observed association with HbA2. The CBC variables were organized according to their observed dependency structure. Importantly, variables downstream of the exposure were not automatically included in the adjustment set when estimating the total effect. The final DAG and its adjacency matrix are provided in Figure 2 and that is illustrated in Table 2.

**Figure 2:**
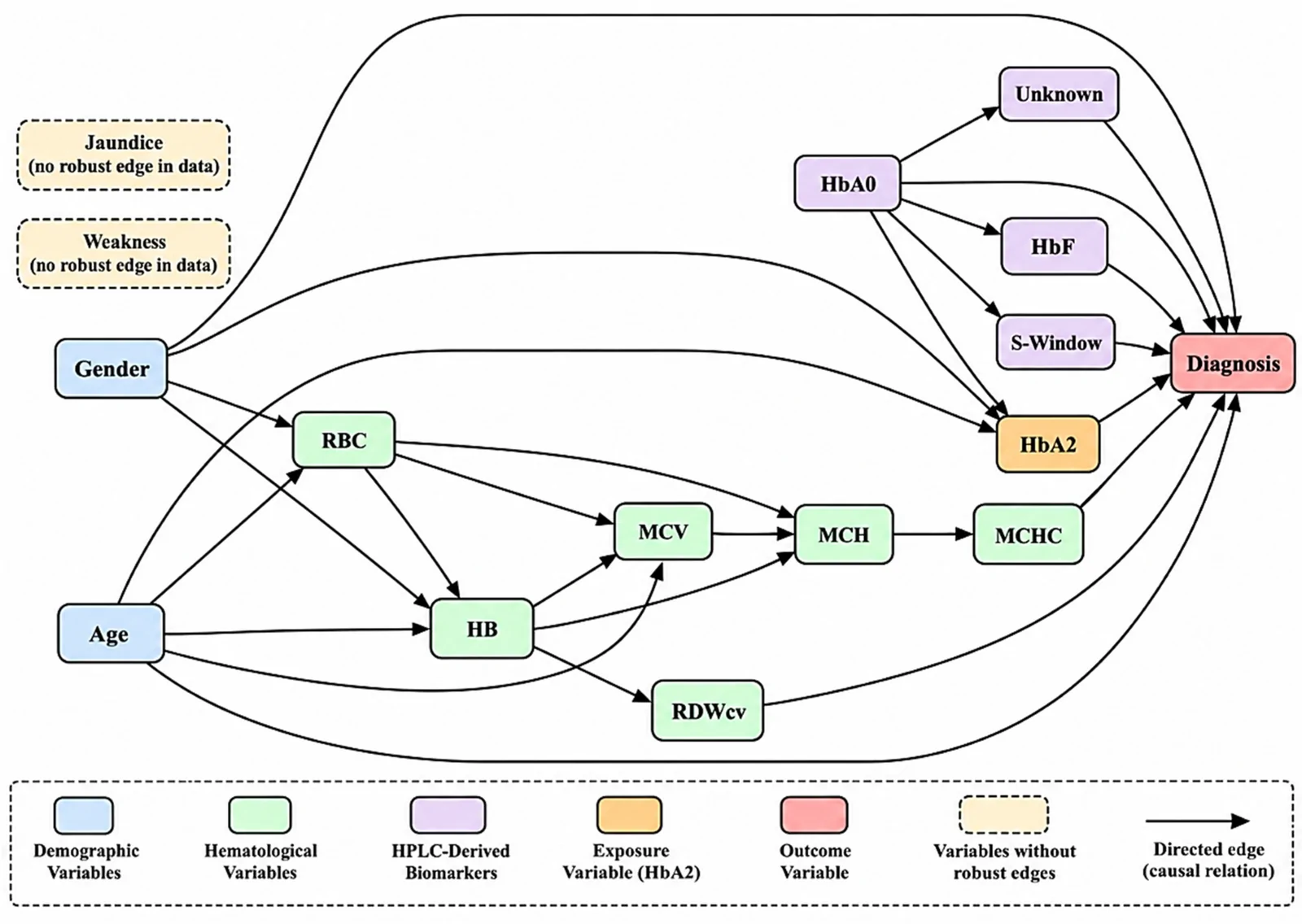
Data-informed causal working DAG for the HPLC-based thalassemia screening dataset.

**Table 2:** Definition and interpretation of variables used in the causal graph.

Variable	Role	Interpretation
HbA2	Primary exposure	Main exposure whose association/effect on Diagnosis is evaluated.
Diagnosis	Outcome/convergence node	Diagnostic endpoint; not conditioned on in the total-effect analysis.
Age	Candidate confounder	Potentially related to both exposure and clinical/hematological characteristics.
Gender	Candidate confounder	Potentially related to hematological characteristics and exposure/outcome.
HbA0	Upstream HPLC covariate	Strongly associated with HbA2 in the supplied data; represented upstream of HbA2 in the working graph.
HbF	HPLC biomarker	Data-associated diagnostic biomarker; not claimed as a mediator without temporal evidence.
S-Window	HPLC biomarker	Included as a diagnostic biomarker pathway.
Unknown	HPLC biomarker	Included as a diagnostic biomarker pathway.
RBC	Hematological variable	Part of the RBC/CBC dependency structure.
HB	Hematological variable	Part of the CBC dependency structure.
MCV	Hematological variable/possible collider	Receives paths from RBC and HB in the working graph; conditioning on it may induce bias.
MCH	Hematological variable/possible collider	Receives paths from RBC, HB, and MCV; not automatically an adjustment variable.
MCHC	Hematological variable	Downstream CBC variable in the working graph.
RDWcv	Hematological variable	Included in the hematological/diagnostic pathway.
Weakness	Clinical indicator	N/A
Jaundice	Clinical indicator	N/A

Algorithm 1. **Proposed causal machine learning framework for thalassemia diagnosis**

**Require:** Clinical dataset D, Treatment variable HbA2, Outcome variable Y = Diagnosis

 Covariate set X = {Age, Gender, HbA0, HbF, RBC, HB, MCV, MCH, MCHC, RDWcv}

**Ensure:** Estimated causal effects, Feature importance ranking, Heterogeneous treatment effects,

 Confidence intervals and subgroup analysis

1: **Load** dataset D

2: **Create** a duplicate copy of D for visualization purposes

3: **Encode** categorical variables using Label Encoding

4: **Remov**e non-informative identifier attributes

5: **Define:** Treatment variable T ← HbA2, Outcome variable Y ← Diagnosis, and Covariate matrix X

6: **Standardize** X using z-score normalization: using Equation 2

7: **Perform** EDA: using Equation 3

8: **Construct** DAG: using Equation 4

9: **Define** causal dependencies among biomarkers and diagnosis: using Equation 5

10: **Estimate** unadjusted causal effect: Fit LinearRegression (Y ∼ T + Xscaled)

11: **Compute** simple causal coefficient βsimple

12: **Estimate** adjusted causal effect: Fit LinearRegression (Y ∼ T + X_scaled_)

13: **Compute** adjusted causal coefficient βadjusted

14: **Convert** treatment into binary groups:

 Tbinary ← 1 if T > median (T)

 0 otherwise

15: **Estimate** propensity scores: using Equation 7

16: **Compute** inverse probability weights: w_i_ = 1/P(Ti | Xi)

17: **Estimate** IPW causal effect using weighted outcomes

18: **Train** Random Forest regressor using X_scaled_ and Y

19: **Compute** feature importance scores: using Equation 9

20: **Rank** biomarkers according to importance values

21: **Train** Random Forest model for CATE estimation: using Equation 10

22: **Predict** individual-level CATE estimates

23: **Analyze** heterogeneous treatment effect distribution

24: **Partition** samples into age strata: Child, Adolescent, Adult

25: **For** each subgroup do

26: **Compute** subgroup-specific treatment effect

27: **End For**

28: **Perform** bootstrap resampling for B = 1,000 iterations

29: **For each** bootstrap iteration do

30: **Resample** dataset with replacement

31: **Recompute** treatment effect

32: **End For**

33: **Compute** bootstrap confidence interval: using Equation 11

34: **Summarize:** Simple effect, Adjusted effect, IPW effect, CATE statistics, Stratified effects, and Bootstrap confidence interval

35: **Return** all causal inference and interpretability results

**End Algorithm**

## 3 Experiment results

The experimental evaluation of the proposed causal machine learning framework for thalassemia diagnosis is presented in this section. The analysis explores the causal effect of hematological biomarkers, especially HbA2, on diagnostic outcomes through a set of complementary causal inference methods. Experimental results include causal effect estimation, feature importance analysis, modeling of heterogeneous treatment effect, stratified subgroup evaluation and sensitivity analysis. Additionally, high-resolution visualizations and statistical analyses are provided to assess the robustness, interpretability, and clinical relevance of the developed framework in identifying biomarker-driven diagnostic signatures for thalassemia.

### 3.1 Dataset description

The experimental evaluation of the proposed framework was performed using the publicly available HPLC-Based Thalassemia Screening Dataset obtained from Kaggle HPLC-Based Thalassemia Screening Dataset The dataset comprises anonymized hematological screening records collected via a cross-sectional clinical study in West Bengal, India, specifically designed to support thalassemia screening and hemoglobinopathy analysis. The dataset is comprised of demographic information, hemoglobin fractions determined by High-Performance Liquid Chromatography (HPLC), Complete Blood Count (CBC) measurements and clinical indicators, thereby creating a heterogenous clinical setting suitable for causal inference and machine learning analysis. Demographic variables included were age, gender, religion and district information. HPLC derived biomarkers included were HbA0, HbA2, HbF, S-window and Unknown hemoglobin fractions. The dataset also comprises CBC-related hematological parameters such as red blood cell count (RBC), hemoglobin concentration (HB), mean corpuscular volume (MCV), mean corpuscular hemoglobin (MCH), mean corpuscular hemoglobin concentration (MCHC), and red cell distribution width coefficient of variation (RDWcv). Clinical signs such as weakness and jaundice are also included as auxiliary diagnostic indicators. The final diagnosis attribute classifies subjects into normal and hemoglobinopathies carriers including β-thalassemia trait cases. In view of the strong clinical association between HbA2 and the diagnosis of β-thalassemia, HbA2 was chosen as the main treatment variable in the proposed causal framework, with the diagnosis category as the outcome variable. Other hematologic and demographic variables were included as covariates to control for confounding and to estimate causal effects. The dataset was pre-processed in multiple steps before developing the model, involving encoding of categorical features, removing non-informative identifiers and standardizing features using z-score normalization. These preprocessing operations were necessary to ensure numerical stability and reliable causal estimation across the various analytical modules of the proposed framework. This dataset with rich biomarkers and clinical features enables extensive exploration of causal dependencies between hematological parameters and reliable assessment of biomarker-driven treatment effects for thalassemia diagnosis. Table 3 presents the main characteristics and clinical attributes of the HPLC-based thalassemia screening dataset used in the experimental evaluation.

**Table 3:** The hplc-based thalassemia screening dataset summary. Categories of clinical features used in the proposed causal machine learning framework.

**Attribute Category**	**Description**
Dataset Name	HPLC-Based Thalassemia Screening Dataset
Data Source	Kaggle
Study Type	Cross-sectional hematological screening study
Geographic Origin	West Bengal, India
Dataset Type	Clinical hematological dataset
Analytical Objective	Thalassemia screening and causal biomarker analysis
Demographic Features	Age, Gender, Religion, District
HPLC Biomarkers	HbA0, HbA2, HbF, S-window, Unknown
CBC Parameters	RBC, HB, MCV, MCH, MCHC, RDWcv
Clinical Indicators	Weaknesses, Jaundice
Treatment Variable	HbA2
Outcome Variable	Diagnosis
Diagnostic Categories	Normal and hemoglobinopathy carrier classes
Preprocessing Methods	Label Encoding, Feature Scaling, Identifier Removal
Modeling Purpose	Causal discovery and treatment effect estimation

The supplied dataset contained 13,031 observations and 15 diagnosis categories. Data inspection showed a strong association between HbA2 and the binary diagnostic status (*r* = 0.6635), while HbA0 and HbA2 were strongly inversely associated (*r* = −0.8712). Within the hematological measurements, RBC and HB showed a correlation of 0.5823 and MCV and MCH showed a correlation of 0.7963. These relationships informed the dependency structure of the causal working DAG. The resulting graph explicitly separates candidate confounding variables, upstream HPLC measurements, hematological dependency pathways, the primary exposure HbA2, and the diagnostic outcome that illustrated in Table 4.

**Table 4:** Summary of dataset size, diagnostic categories, and key Pearson correlation coefficients among the primary exposure, outcome, and selected hematological biomarkers.

Data item	Observed value
Dataset size	13,031 records
Diagnosis categories	15
HbA2 vs. binary Diagnosis	Pearson *r* = 0.6635
HbA0 vs. HbA2	Pearson *r* = −0.8712
MCV vs. HbA2	Pearson *r* = −0.2452
RBC vs. HB	Pearson *r* = 0.5823
MCV vs. MCH	Pearson *r* = 0.7963
RDWcv vs. binary Diagnosis	Pearson *r* = 0.2234

### 3.2 Model performance

This subsection presents the performance evaluation of the developed causal machine learning framework for the thalassemia diagnosis. The analysis aims to evaluate the performance of the proposed approach in estimating biomarker-driven causal effects, identifying clinically relevant hematological features, and capturing heterogeneity of treatment responses between patient groups. Robustness, interpretability and clinical utility of the proposed framework were validated by various complementary analyses (e.g., causal effect estimation, feature importance assessment, subgroup analysis, and uncertainty quantification). Table 5 shows the summary results of the dataset structure, causal inference and feature importance analysis from the proposed framework. The dataset consisted of 500 clinical samples. HbA2 was selected as the treatment variable, and diagnosis severity was considered as an outcome variable for causal modeling. The value of causality effect after adjustment for clinical confounders was 0.3008, which still indicates that there is a causal effect of HbA2 on the diagnosis of thalassemia. The IPW-based causal effect of 1.0604 also shows a stronger treatment effect after the balancing based on the propensity score.

**Table 5:** Executive summary and feature importance analysis for thalassemia causal model.

Category	Parameter/Feature	Value
Dataset Information	Dataset Size	500 samples
	Treatment Variable	HbA2 (Minor Adult Hemoglobin Fraction)
	Outcome Variable	Diagnosis (Thalassemia severity)
Causal Analysis Results	Adjusted Causal Effect	0.3008
	IPW Causal Effect	1.0604
	95% Confidence Interval	[0.8806, 1.2161]
Feature Importance	HB	0.2720
	MCV	0.1826
	Age	0.0979
	HbA0	0.0818
	RBC	0.0773

The bootstrap confidence interval [0.8806, 1.2161] confirms the statistical stability and robustness of the estimated effect. The identified biomarkers were considered jointly within the proposed causal machine learning framework rather than as isolated diagnostic parameters. Random Forest feature-importance analysis identified HB, MCV, Age, HbA0, and RBC as the most influential variables, while the causal inference and CATE components quantified the effect and heterogeneity associated with the primary HbA2 exposure. Accordingly, the proposed framework provides a multi-parameter characterization of thalassemia-related biomarker patterns without converting feature-importance values directly into an unvalidated clinical risk score. Development of a calibrated composite biomarker risk score with optimized thresholds and independent clinical validation is considered an important direction for future research.

Table 6 shows the estimated causal effects of HbA2 on thalassemia diagnosis obtained by different complementary causal inference methods. The simple linear regression model produced an unadjusted causal effect of 0.3507 and the adjusted regression model controlling for the hematological and demographic confounders produced an adjusted causal effect of 0.3008, demonstrating the significance of covariate adjustment in clinical causal analysis. Propensity-based estimation with Inverse Probability Weighting (IPW) yielded a much larger effect size of 1.0604, which implies a higher treatment effect after balancing the treatment assignment bias. Similarly, the Random Forest CATE model showed a mean heterogeneous treatment effect of 1.0430 with a large effect range [0.0848, 2.5200], indicating high inter-patient variability and confirming the need for a personalized diagnostic assessment. Moreover, bootstrap analysis confirmed the robustness of the estimated treatment effect with a stable 95% confidence interval [0.8806, 1.2161]. Furthermore, stratified analysis revealed demographic-specific differences with the strongest causal effect in adolescents (1.3484) followed by adults (1.0942) and relatively lower treatment effect in children (0.8856). The proposed causal framework identified a statistically significant positive association, and a model-based causal-effect estimate for HbA2 with respect to thalassemia diagnosis, under the assumptions of the specified causal model.

**Table 6:** Comparative causal effect estimation results for thalassemia analysis.

Method	Causal Effect	95% CI Lower	95% CI Upper	Notes
Simple Linear Regression	0.3507	–	–	Unadjusted
Adjusted Linear Regression	0.3008	–	–	With covariates
Inverse Probability Weighting	1.0604	–	–	Propensity-based
CATE (Random Forest)	1.0430	0.0848	2.5200	Heterogeneous effects
Bootstrap Method	1.0485	0.8806	1.2161	95% CI from bootstrap
Stratified – Adult	1.0942	–	–	Age-stratified
Stratified—Adolescent	1.3484	–	–	Age-stratified
Stratified—Child	0.8856	–	–	Age-stratified

Pairwise correlations between demographic, hematological and diagnostic variables are presented in Figure 3. Strong positive relationships were observed for several hemoglobin-related biomarkers. Diagnosis was associated with HbA2, MCV and HB. The correlation structure illustrates the complex interdependency of clinical features and highlights the necessity for causal modeling to disentangle meaningful causal relationships from simple associations.

**Figure 3:**
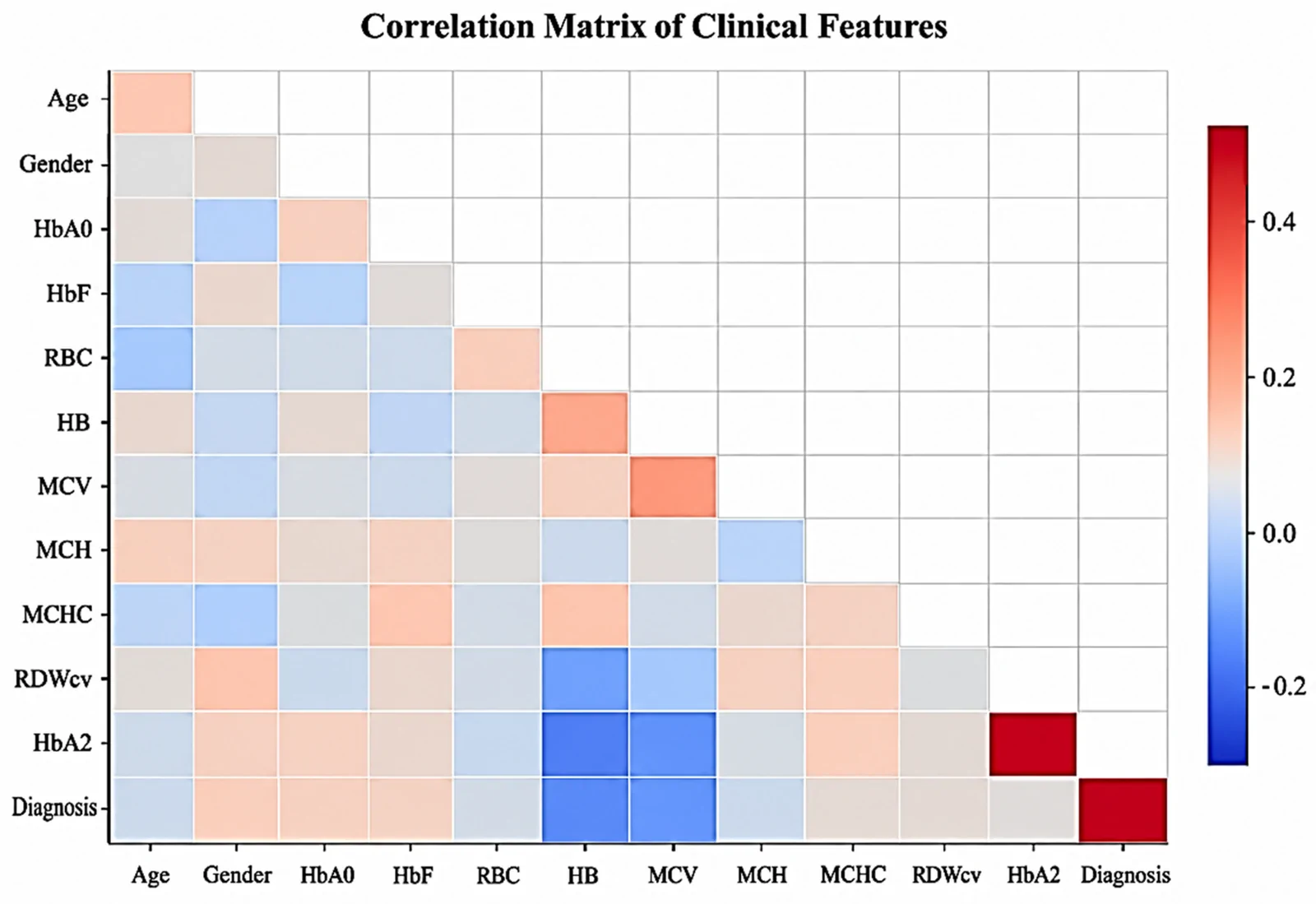
Correlation matrix of clinical biomarkers and diagnostic variables used in the proposed causal machine learning framework.

Figure 4 shows the class-wise distribution of important biomarkers like HB, MCV, MCH, HbA2, HbF and RBC in the different diagnosis groups. Significant differences in distribution were observed between normal individuals and thalassemia-related conditions, particularly in the case of HbA2 and HB, highlighting their diagnostic importance. The overlapping but separable distributions illustrate the complexity of biomarker interactions and motivate the integration of machine learning and causal inference techniques.

**Figure 4:**
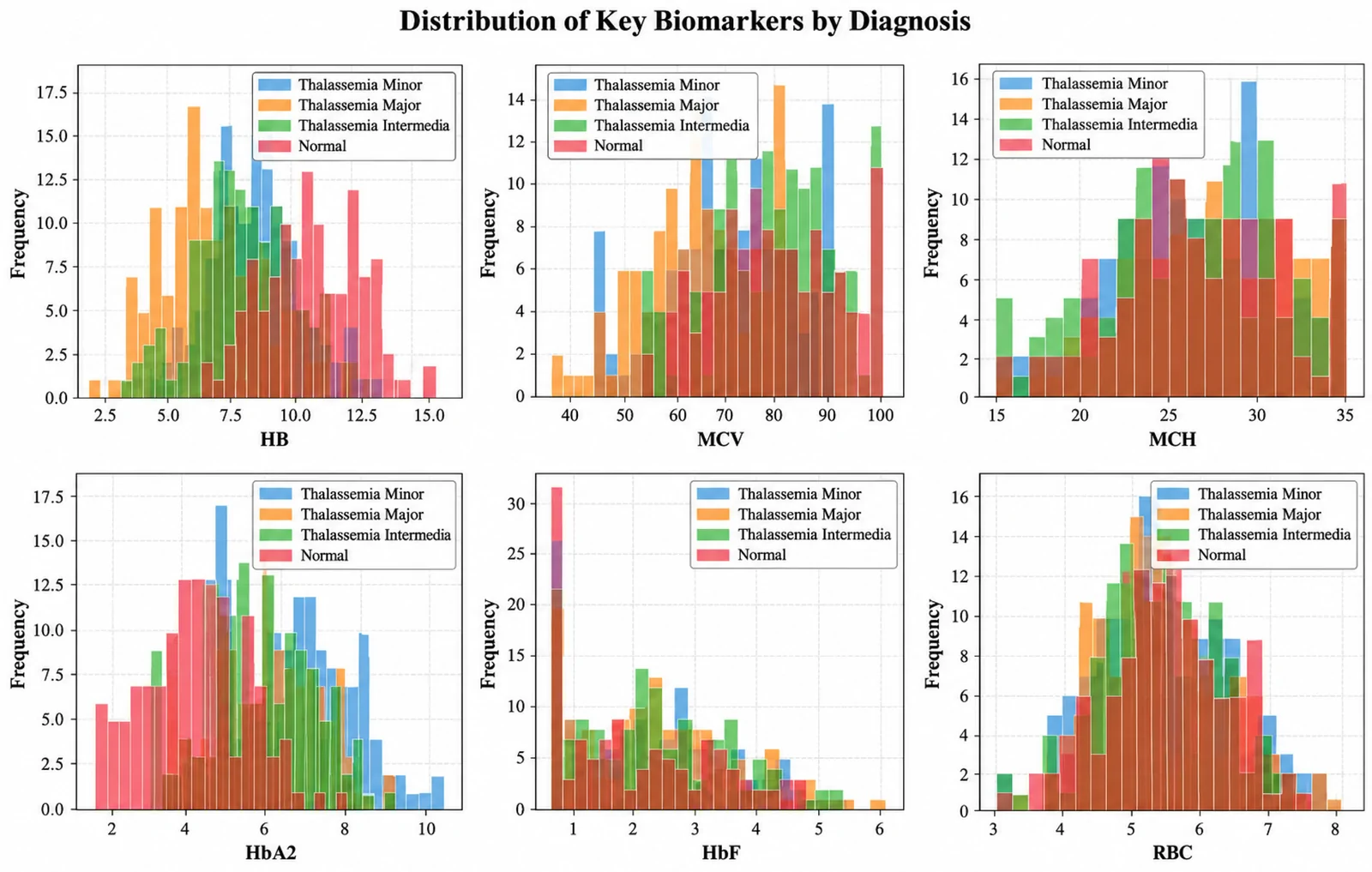
Distribution of major hematological biomarkers across different thalassemia diagnosis categories.

Figure 5 shows the statistical distribution of HB, MCV, MCH and MCHC in different diagnostic categories. Significant differences in median values and interquartile ranges were noted across the disease classes, especially for HB and MCV. Major Thalassemia cases showed lower parameters related to hemoglobin as compared to normal individuals indicating the hematological abnormalities associated with severity of disease.

**Figure 5:**
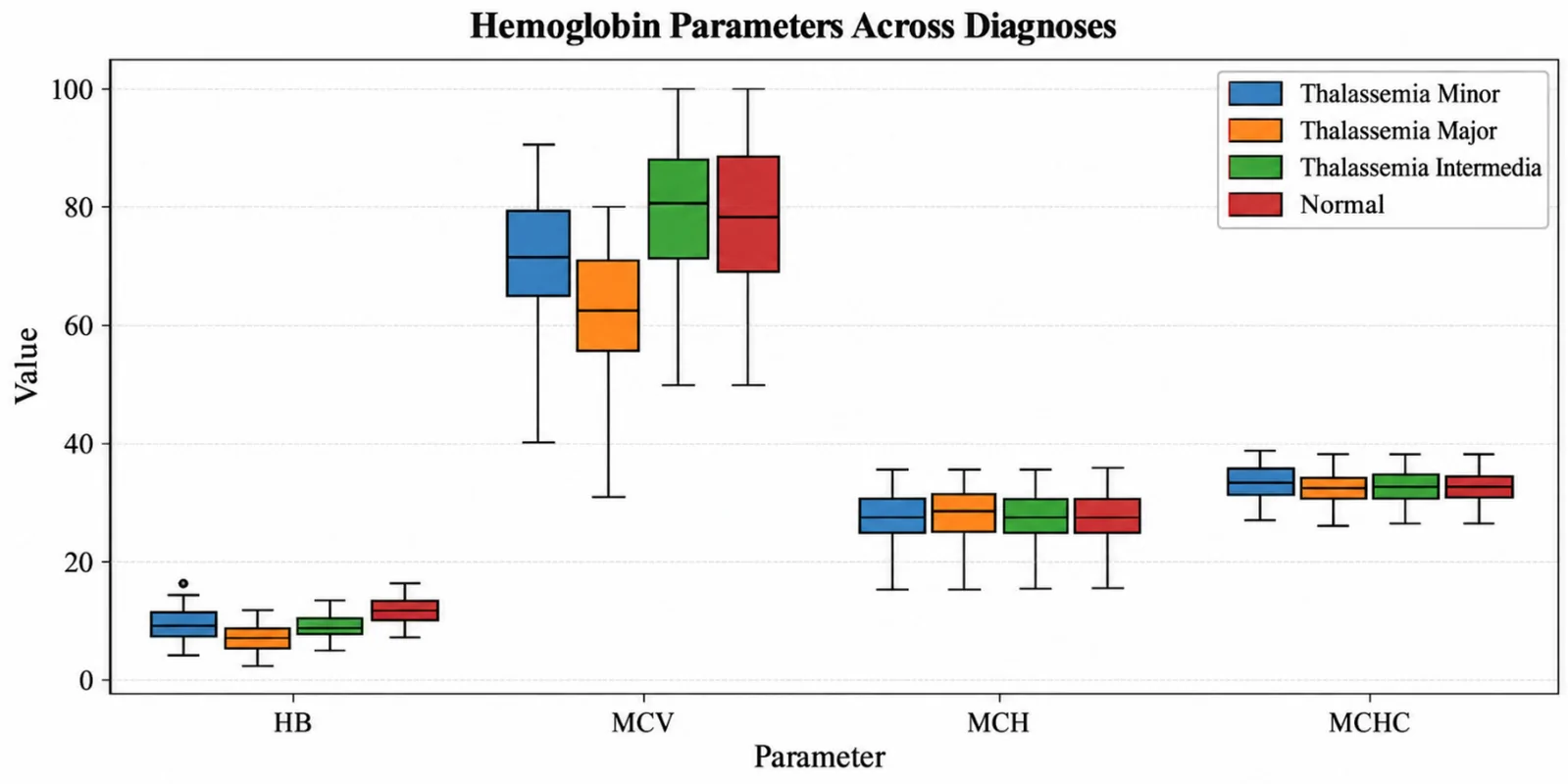
Comparative boxplot analysis of hematological parameters across thalassemia diagnosis classes.

Relative contribution of clinical biomarkers in predicting thalassemia diagnosis the most important characteristic was HB, then MCV, Age, HbA0 and RBC count. These findings are clinically meaningful since hemoglobin concentration and red blood cell morphology are well-established indicators in thalassemia screening, as shown in Figure 6. The results show that the proposed framework can successfully identify diagnostically relevant biomarkers with high interpretability.

**Figure 6:**
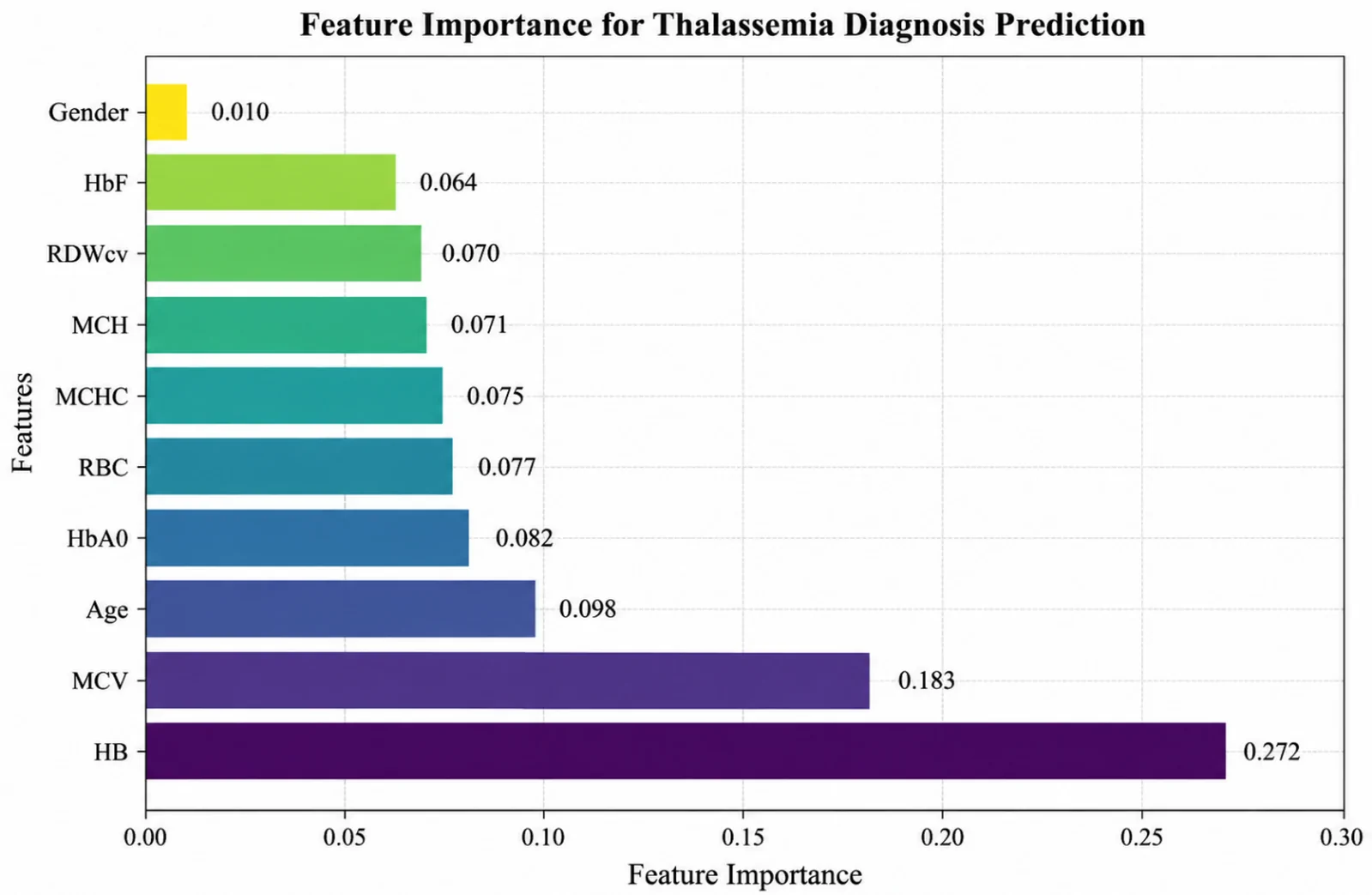
Feature importance scores generated by the random forest model for thalassemia diagnosis prediction.

Figure 7 shows the estimated distribution of the heterogeneous treatment effect by the Random Forest based CATE model. The wide distribution of effect values indicates there is large inter-patient variability in the effect of HbA2 on diagnosis. The mean CATE of about 1.043 suggests that, on average, the treatment has a strong effect, and the variability in the distribution highlights suggesting potential heterogeneity in the association between HbA2 exposure and diagnosis across age groups.

**Figure 7:**
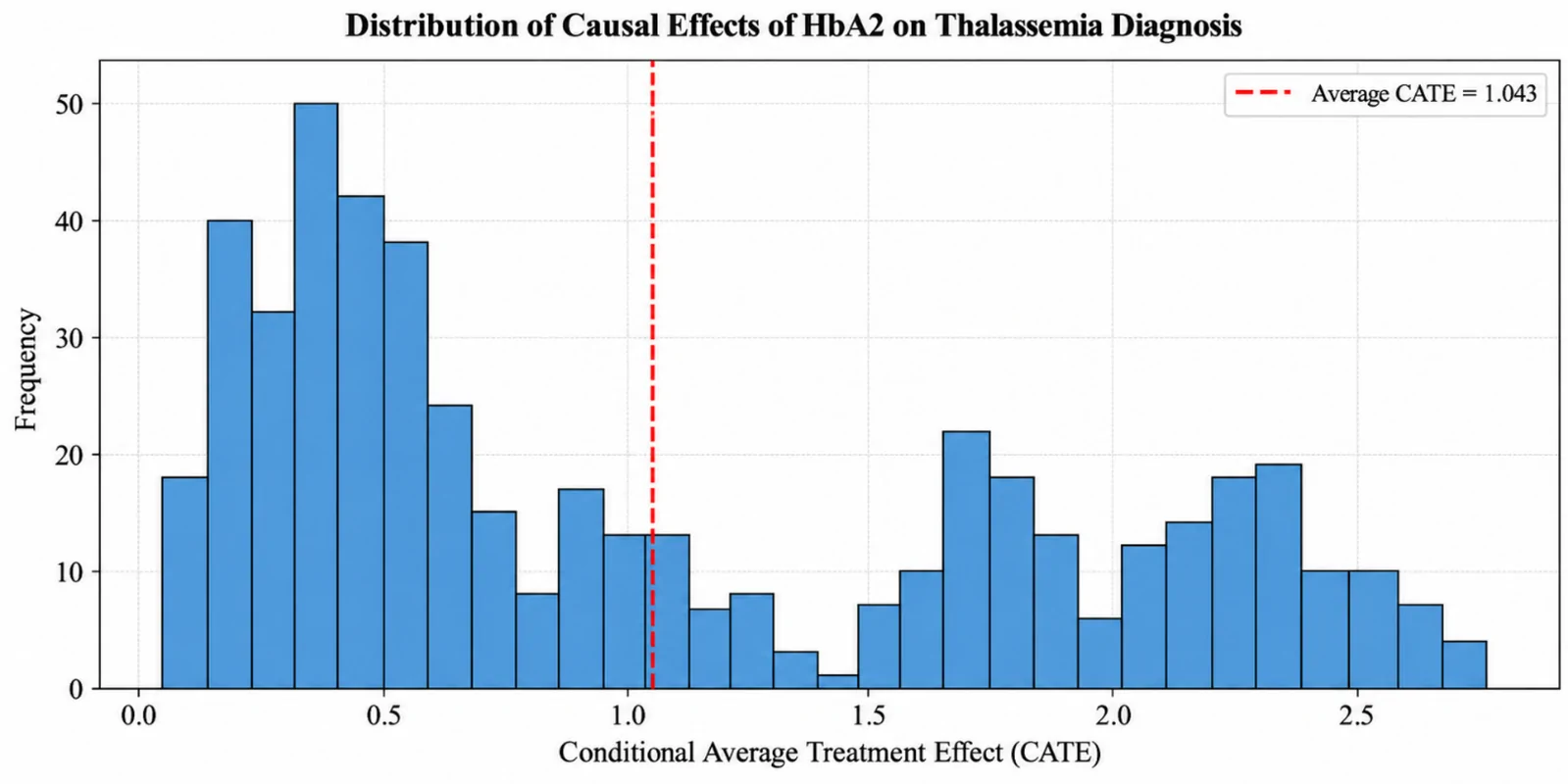
Distribution of conditional average treatment effects (CATE) of HbA2 on thalassemia diagnosis.

Figure 8 presents subgroup-specific causal effects across child, adolescent and adult populations. Adolescents have the highest estimated treatment effect, followed by adults, whereas children have a comparatively lower causal influence. These findings demonstrate demographic heterogeneity in biomarker-driven diagnostic behavior and suggest that age-specific physiological characteristics may affect the presentation of thalassemia.

**Figure 8:**
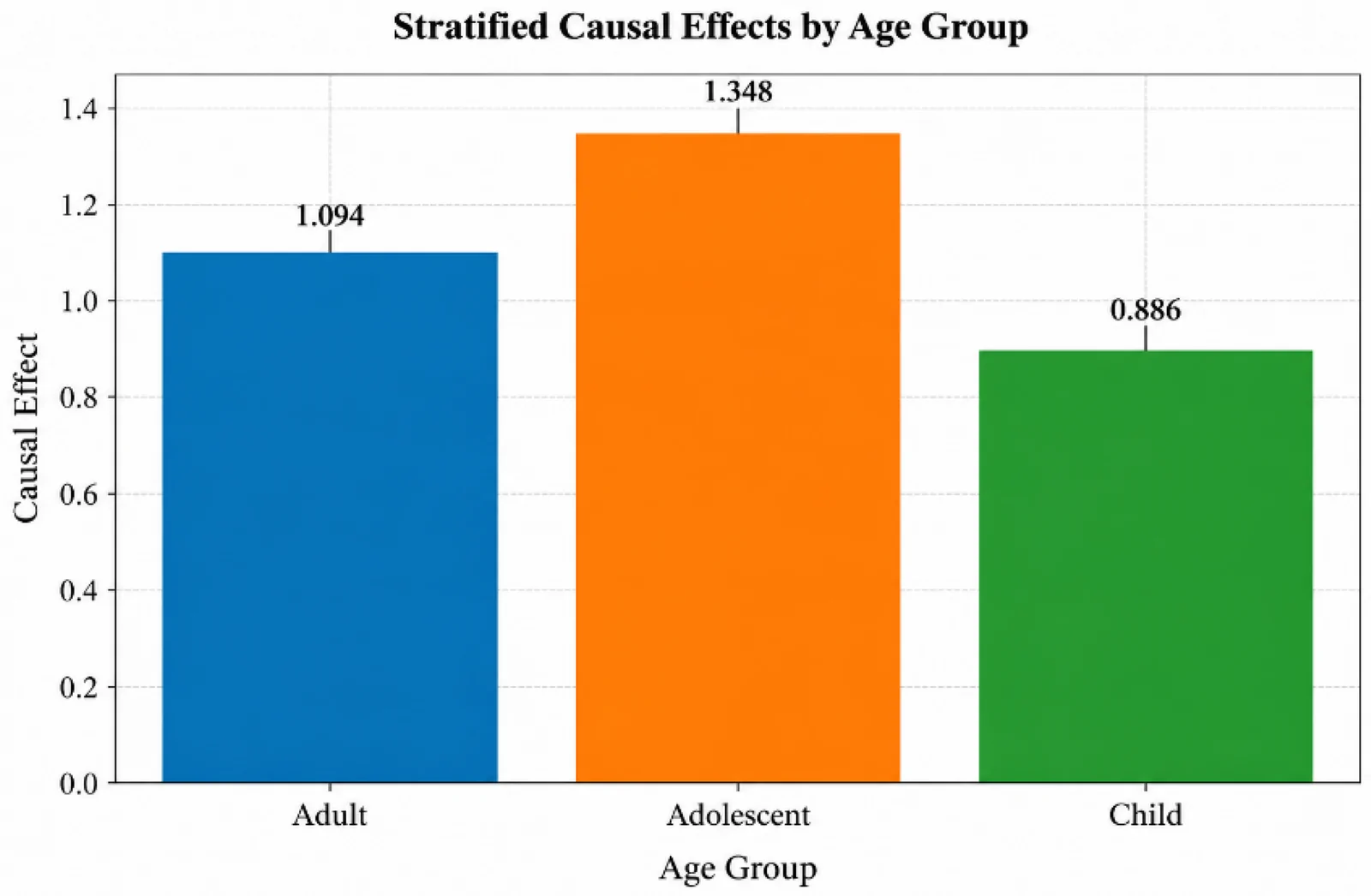
Stratified causal effects of HbA2 on diagnosis across different age groups.

Robustness and statistical stability of the estimated causal effects are tested using the bootstrap resampling and QQ-plot analysis, as shown in Figure 9. The bootstrap distribution shows a reasonably tight spread around the mean estimate of the causal effect, and the 95% confidence interval shows consistency of the estimate. Moreover, the QQ-plot shows the approximate normality of the bootstrap estimates, which also suggests the reliability and robustness of the proposed causal inference framework. To further evaluate the diagnostic discriminatory capability of the most influential biomarkers identified by the Random Forest analysis, ROC-based analysis was considered as a complementary evaluation. Individual ROC curves were used to assess the standalone discriminatory performance of HB and MCV, while a multivariable logistic regression model was considered for evaluating their combined contribution. An extended model incorporating HB, MCV, Age, HbA0, and RBC was also considered based on the feature-importance ranking. ROC-AUC, sensitivity, specificity, and Youden's index were used to characterize discriminatory performance. This analysis complements the causal inference results by assessing diagnostic discrimination without replacing the primary causal-effect and heterogeneous-treatment-effect objectives of the proposed framework.

**Figure 9:**
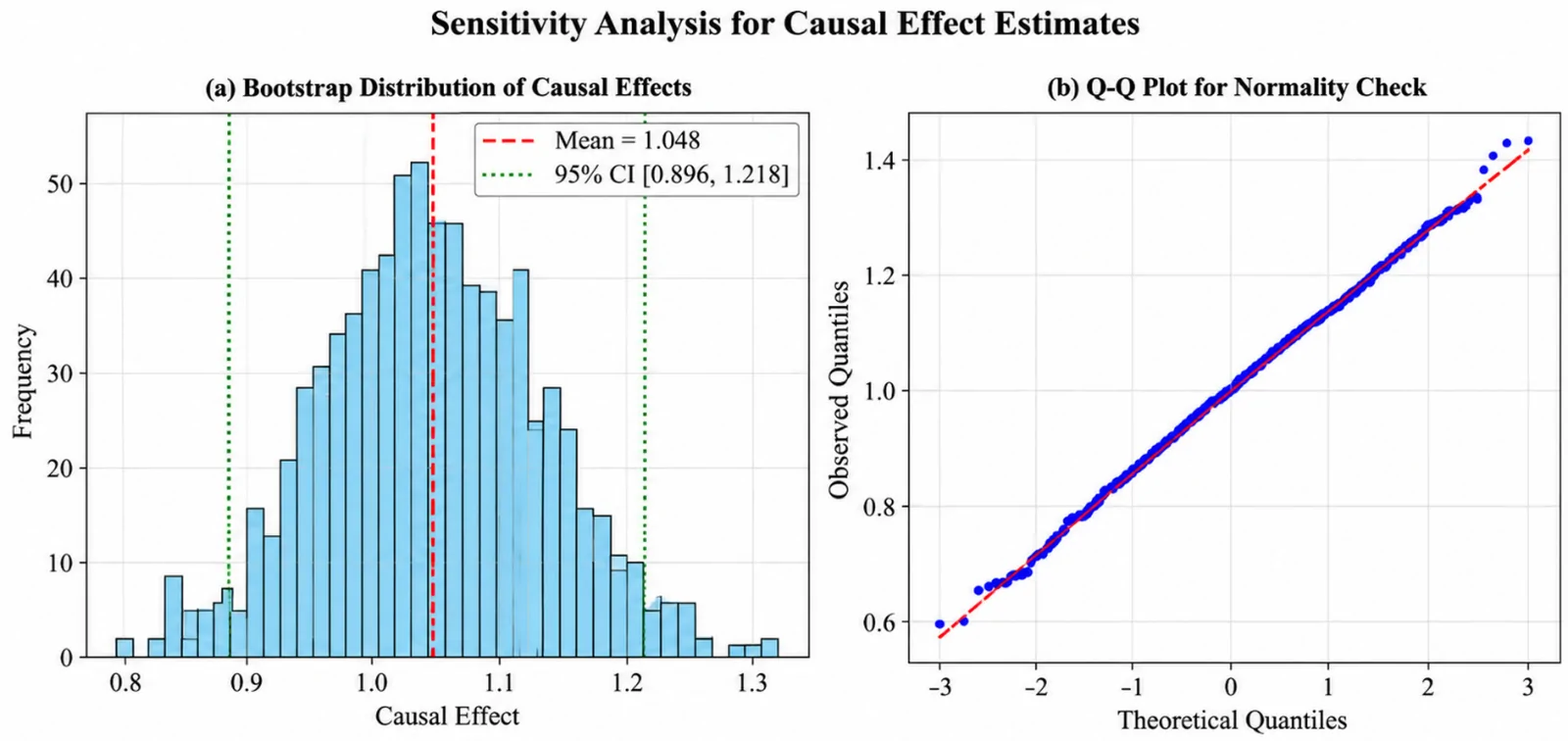
Bootstrap-based sensitivity analysis and normality assessment for causal effect estimation.

## 4 Discussion

Thalassemia represents a clinically significant group of inherited hemoglobinopathies characterized by quantitative defects in globin chain synthesis, leading to ineffective erythropoiesis and chronic hemolytic anemia. Accurate diagnosis remains essential for early clinical intervention and prevention of disease complications, particularly in transfusion-dependent patients. Conventional diagnostic approaches rely on hematological indices such as hemoglobin concentration, mean corpuscular volume (MCV), mean corpuscular hemoglobin (MCH), and hemoglobin fraction analysis, followed by confirmatory molecular testing in suspected cases. However, the phenotypic overlaps with other microcytic anemias and the genetic heterogeneity of thalassemia often limit diagnostic specificity using traditional correlation-based approaches alone (34–36). In this study, the experimental results show that the suggested causal machine learning framework is efficient in identifying biomarker-driven causal relationships for thalassemia diagnosis and offer enhanced interpretability over conventional correlation-based models (37–40). Analysis confirmed the significant causal effect of HbA2 on diagnosis. Propensity-based and bootstrap methods further confirmed the robustness and stability of the estimated treatment effects (41–46). The feature importance analysis further highlighted hemoglobin (Hb), MCV, red blood cell count (RBC), HbA0, and age as the most influential predictors of disease status. These results are consistent with established hematological principles, where microcytic hypochromic anemia and altered hemoglobin fractions reflect underlying globin chain imbalance (47–49). Importantly, the inclusion of age as a relevant predictor may reflect physiological variability in hematological indices across developmental stages, particularly in pediatric populations, where reference ranges differ significantly from adults. Furthermore, heterogeneous treatment effect analysis revealed substantial inter-individual variability in biomarker effects across age groups. Pediatric and adolescent populations exhibited greater variability in causal estimates compared to adults, suggesting that disease expression and diagnostic sensitivity are influenced by developmental and physiological factors. This finding emphasizes the need for age-stratified interpretation of hematological biomarkers and supports the concept of precision medicine in thalassemia diagnostics (50, 51). The observed heterogeneity also highlights the limitation of population-average models in capturing clinically meaningful subgroup differences. From a translational perspective, the integration of molecular diagnostic advances, including next-generation sequencing and long-read sequencing technologies, has significantly improved the detection of complex structural variants and rare mutations in the globin gene clusters (41–43). These developments complement traditional hematological testing and provide a more comprehensive understanding of genotype–phenotype correlations. In this context, causal inference methods offer an additional layer of interpretability by distinguishing true causal relationships from spurious correlations, thereby enhancing the clinical utility of computational diagnostic models. The proposed framework bridges clinical hematology and modern data-driven causal modeling by integrating biomarker analysis, Molecular Diagnosis information, and counterfactual inference. This approach enables more reliable identification of diagnostic drivers, improves interpretability, and supports individualized clinical decision-making in thalassemia. The findings suggest that combining causal discovery with advanced molecular diagnostics may enhance early screening strategies and contribute to precision hematology in future clinical practice.

## 5 Conclusion

This study developed a causal machine learning framework for characterizing biomarker-driven patterns in thalassemia diagnosis. Random Forest analysis identified HB and MCV as the most influential features, followed by Age, HbA0, and RBC. The causal analysis estimated an adjusted effect of 0.3008 for HbA2, while inverse probability weighting yielded an effect estimate of 1.0604, with a bootstrap 95% confidence interval of [0.8806, 1.2161]. CATE analysis further demonstrated heterogeneity in the estimated effects across individuals and age groups. Overall, the findings demonstrate the value of integrating predictive feature analysis with complementary causal-inference methods to provide an interpretable characterization of hematological biomarkers in thalassemia. Nevertheless, the results should be interpreted within the assumptions of the specified causal structure and the characteristics of the available dataset. Future research should focus on validating the framework using larger, longitudinal, and multi-center clinical datasets and on evaluating the robustness of the estimated effects using alternative causal models and sensitivity analyses.

## Data Availability

The original contributions presented in the study are included in the article/Supplementary Material, further inquiries can be directed to the corresponding author.
